# *Streptococcus bovis* – unusual etiology of meningitis in a neonate with Down syndrome: a case report

**DOI:** 10.1186/s13256-018-1634-y

**Published:** 2018-04-12

**Authors:** Sachith Mettananda, Phirarthana Kamalanathan, K. Dhananja Namalie

**Affiliations:** 1 Department of Paediatrics, Faculty of Medicine, University of Kelaniya, Thalagolla Raod, Ragama, 11010 Sri Lanka; 2 Colombo North Teaching Hospital, Ragama, 11010 Sri Lanka

**Keywords:** *Streptococcus bovis*, Down syndrome, Meningitis

## Abstract

**Background:**

Common etiological agents of neonatal meningitis include group B *Streptococcus*, *Escherichia coli,* and *Staphylococcus aureus*. Here we report a rare pathogen – *Streptococcus bovis* – causing meningitis in a premature neonate with Down syndrome.

**Case presentation:**

A 26-day-old Asian male neonate with Down syndrome presented with a history of high-grade fever, poor sucking, poor cry, and reduced activity. On admission, he was febrile and had features of circulatory collapse. A cerebrospinal fluid examination confirmed bacterial meningitis and blood culture isolated the causative organism: group D *Streptococcus*, which was verified as *Streptococcus bovis* biotype 2. An echocardiogram did not show evidence of infective endocarditis.

**Conclusions:**

This is probably the first report of neonatal meningitis due to *Streptococcus bovis* in a child with Down syndrome. Although our patient did not show features of overt immunodeficiency, subtle abnormalities in his immune system would have predisposed him to infection with this unusual organism. This case highlights the need for considering unusual pathogens when managing serious infections in children with Down syndrome.

## Background

Neonatal meningitis is a serious infection of the central nervous system which results in significant morbidity and mortality [1]. Common etiological agents are *Streptococcus agalactiae* (group B *Streptococcus*), *Staphylococcus aureus,* and *Escherichia coli* worldwide. Rare pathogens that cause neonatal meningitis include coagulase-negative *Staphylococci*, *Pseudomonas*, *Klebsiella, Enterococcus,* and *Listeria monocytogenes;* however, they are mostly seen in neonates with congenital or acquired immunodeficiency [2]. Here we report a rare pathogen – *Streptococcus bovis* – causing meningitis in a premature neonate with Down syndrome. This case highlights the importance of considering rare and unusual pathogens when encountered with severe infections in children with Down syndrome.

## Case presentation

A 26-day-old Asian male neonate with Down syndrome presented with a history of high-grade fever, poor sucking, poor cry, and reduced activity of 1-day duration. He was born by emergency lower segmental cesarean section to a 43-year-old primigravida mother at 34 weeks of gestation with a birth weight of 1.68 kg. Antenatal history was complicated with pregnancy-induced hypertension and reversed diastolic flow in umbilical arteries during third trimester. The baby had been exclusively breast-fed since birth and did not have vomiting or diarrhea.

On examination, he was febrile, 38.3 °C (101 °F), and had mottled skin. His pulse rate was 170 beats per minute, with prolonged capillary refilling time and cold peripheries; his blood pressure was 89/53 mmHg. Precordial evaluation revealed dual rhythm and a grade 3 ejection systolic murmur at the pulmonary area. His respiratory rate was 50/minute with signs of moderate respiratory distress. His anterior fontanel was flat, both pupils were equally reactive to light, and examinations of spine and extremities were normal. A provisional diagnosis of septic shock was made and initial resuscitation with supplementary oxygen and normal saline bolus was carried out; intravenously administered flucloxacillin and cefotaxime were commenced.

A complete blood count revealed total leukocytes 6500/mm^3^ (neutrophils 72%, lymphocytes 17%, and monocytes 9%), hemoglobin 13.2 g/dl, and platelet count 349,000/mm^3^. Random blood glucose was 123 mg/dl and C-reactive protein level was 38.6 mg/L. A lumbar puncture was performed 4 hours after the first dose of antibiotics and cerebrospinal fluid (CSF) analysis revealed: leukocytes 11/mm^3^ (ten polymorphonuclear cells and one lymphocytes), protein 124 mg/dL, and glucose 52 mg/dL. Blood culture yielded growth of group D *Streptococcus*, which was verified as *Streptococcus bovis* biotype 2 by manual analysis (demonstrating growth in MacConkey broth agar and Bile Esculin agar and absent growth in 6.5% sodium chloride medium) and by the automated bacterial identification technology. The organism was sensitive to penicillin and ampicillin; therefore, flucloxacillin was replaced by intravenously administered benzylpenicillin. CSF Gram stain demonstrated no organisms and cultures of CSF were negative probably due to antibiotic administration prior to lumbar puncture. Two-dimensional echocardiography revealed a small ostium secundum atrial septal defect without evidence of infective endocarditis. Karyotype confirmed trisomy 21.

Intravenously administered benzylpenicillin and cefotaxime were continued for 21 days during which the baby showed complete recovery. There were no neurological complications during acute or convalescent periods. The follow-up after 6 months confirmed normal neurological examination except for hypotonia due to Down syndrome and normal occipitofrontal circumference.

## Discussion

*Streptococcus bovis* is a Gram-positive, Lancefield group D *Streptococcus* which is found in the pharynx, nose, and gastrointestinal tract of healthy humans [3]. It is an unusual etiology of human diseases; however, it is known to cause infective endocarditis in hosts with predisposing cardiac defects [4]. Meningitis due *Streptococcus bovis* is rare and is reported in patients with immunodeficiency, cancer, or colon diseases [5–7].

The neonate described in this case report has Down syndrome; however, he did not show other features of primary or secondary immunodeficiency. He had an atrial septal defect but was neither predisposed to nor had infective endocarditis. To the best of our knowledge this is the first case report of neonatal meningitis due to *Streptococcus bovis* in a child with Down syndrome. Previous reports have shown that children with Down syndrome have intrinsic defects in the immune system and are at higher risk of developing infections due to *streptococcal* organisms [8, 9]. Abnormalities in the immune system associated with Down syndrome include reduced T lymphocyte and B lymphocyte counts, absence of normal lymphocyte expansion in infancy, impaired mitogen-induced T cell proliferation, reduced specific antibody responses to immunizations, and defects of neutrophil chemotaxis [10]. We believe that the presence of Down syndrome in this baby would have predisposed him to the severe infection with this unusual organism.

Alternatively, the infection by *Streptococcus bovis* could be related to the premature birth of this baby. Immune system components, both innate and adaptive, show distinct deficits in premature babies which include deficient skin barrier function, fewer commensals in intestinal microbiome, inefficient production of complement proteins, limited neutrophil storage pool, reduced expression of CD40 ligand, and inefficient class switching from IgM to other antibody isotypes [11]. These deficits may also have contributed to infection by *Streptococcus bovis* in this child.

Another important fact about *Streptococcus bovis* infection is its association with colorectal cancer. Particularly in adults, *Streptococcus bovis* bacteremia and endocarditis are associated with colon cancer, neoplastic colon polyps, and other gastrointestinal malignancies [12]. The significance of these associations is not yet known and it is not clear whether infections are causative factors or results of these malignancies. None of the reported cases of pediatric patients with *Streptococcus bovis* infections show associations with colonic diseases; therefore, this association may be limited to adults.

## Conclusions

In conclusion, this case report adds to the medical literature of causative organisms of neonatal meningitis in an apparently immunocompetent child with Down syndrome. It also highlights the need for considering unusual pathogens when managing severe infections in children with Down syndrome.
